# Anti-Trop2 blockade enhances the therapeutic efficacy of ErbB3 inhibition in head and neck squamous cell carcinoma

**DOI:** 10.1038/s41419-017-0029-0

**Published:** 2018-01-05

**Authors:** Nathan Redlich, Anthony M. Robinson, Kwangok P. Nickel, Andrew P. Stein, Deric L. Wheeler, Douglas R. Adkins, Ravindra Uppaluri, Randall J. Kimple, Brian A. Van Tine, Loren S. Michel

**Affiliations:** 1 Medical College of Wisconsin, Milwaukee, WI USA; 2 Washington University in St. Louis School of Medicine, St. Louis, MO USA; 3 Department of Human Oncology, University of Wisconsin School of Medicine and Public Health, and University of Wisconsin Carbone Cancer Center, Madison, WI USA; 4 School of Medicine, Department of Otolaryngology, Case Western Reserve University, Cleveland, OH USA; 5 Dana Farber Cancer Institute, Boston, MA USA; 6 Memorial Sloan-Kettering Cancer Center, Monmouth, NJ USA

## Abstract

ErbB3 has been widely implicated in treatment resistance, but its role as a primary treatment target is less clear. Canonically ErbB3 requires EGFR or ErbB2 for activation, whereas these two established treatment targets are thought to signal independently of ErbB3. In this study, we show that ErbB3 is essential for tumor growth of treatment-naive HNSCC patient-derived xenografts. This ErbB3 dependency occurs via ErbB3-mediated control of EGFR activation and HIF1α stabilization, which require ErbB3 and its ligand neuregulin-1. Here, we show that ErbB3 antibody treatment selects for a population of ErbB3-persister cells that express high levels of the transmembrane protein Trop2 that we previously identified as an inhibitor of ErbB3. Co-treatment with anti-ErbB3 and anti-Trop2 antibodies is synergistic and produces a greater anti-tumor response than either antibody alone. Collectively, these data both compel a revision of ErbB-family signaling and delineate a strategy for its effective inhibition in HNSCC.

## Introduction

Head and neck squamous cell cancer (HNSCC) is a collection of diseases arising from the mucosal surfaces of the oral cavity, oropharynx, nasopharynx, hypopharynx, and larynx. With the exception of oropharyngeal cancers, which are now commonly caused by human papilloma virus, and nasopharyngeal cancers, which are often caused by Epstein-Barr virus, most of these tumors are smoking related^1–3^. Tobacco smoke produces a significant mutational burden in smoking related cancers such as HNSCC and other aerodigestive tumors, and is presumed to be responsible for transformation^4–6^. However, similar to other smoking related tumors, HNSCC sequencing efforts have revealed that mutations are often scattered throughout the genome, and the number of high-frequency actionable mutations (i.e., therapeutically targetable) is limited. This situation lies in contrast to virally related oropharynx cancer, where the catalytic unit of PI3 kinase is mutated in approximately thirty percent of cases^7–9^. Recurrent disease after curative therapy may be associated with an increasing set of mutational events, but the magnitude of these changes remains to be extensively investigated^10^, and validated treatment targets are in great need for HNSCC.

Epidermal growth factor receptor (EGFR) is the only validated treatment target in HNSCC, and it is the most commonly overexpressed oncogene in HNSCC^11^. Targeting EGFR with Cetuximab in combination with radiation increases cure rates by ten percent, and prolongs survival in metastatic disease^12,13^. The other ErbB family members are thought to be involved in HNSCC but only preliminary in vivo investigations of family targeting have been reported^14,15^. ErbB2 (aka HER2) is amplified in HNSCC at a very low frequency and ErbB3 (aka HER3), the kinase-dead member of the family, is neither mutated nor amplified in this disease^11^. ErbB3 has gained attention as a common mechanism of resistance to EGFR-targeted therapies^16–18^. Its activation is dependent on heterodimerization with EGFR or ErbB2, a requirement that lies in contradistinction to the independence of EGFR, for which homodimerization is sufficient to elicit its potent tyrosine kinase activity. However, once ErbB3 heterodimerizes, its six PI3 kinase docking sites can potently drive the PI3 kinase pathway rendering tumors resistant to EGFR-targeted therapies and other conventional agents.

Most pre-clinical studies implicate ErbB3 upregulation in the context of drug resistance^16,17,19–21^ rather than tumorigenesis. Mouse modeling has produced conflicting results in terms of an essential role for ErbB3 in tumor initiation, and the function of ErbB3 appears to be dependent on tissue and initiating oncogene^22–24^. Only in the case of ErbB3 mutation, which is restricted to a small percentage of gastrointestinal carcinomas, has this receptor been found to be intrinsically oncogenic^25^. Therapeutic targeting of ErbB3 in pre-clinical experiments also reveals equivocal results in terms of the anti-tumor and anti-proliferative efficacy of ErbB3 blockade. In HNSCC (and several other tumors), antibody-mediated ErbB3 targeting has been most potent when combined with EGFR or other receptor tyrosine kinase inhibition^26–32^. Moreover, a recent clinical study of combined EGFR and ErbB3 antibodies failed to show improved efficacy compared to single EGFR inhibition with cetuximab^33^. Therefore, as an EGFR-driven tumor, the role of ErbB3 in HNSCC is somewhat unclear.

We previously identified Trop2 as an inhibitor of ErbB3. Trop2 is a multi-functional transmembrane protein with diverse signaling properties^29,34–37^. We reported that Trop2 binds the ErbB3-ligand neuregulin-1, blocking its cleavage and suppressing ErbB3 activation. RNAi-mediated Trop2 loss in HNSCC cell lines not only triggered ErbB3 hyperactivation, but resulted in sensitivity to anti-ErbB3 antibodies. These findings led us to hypothesize that low Trop2 expression is required for optimal sensitivity to anti-ErbB3 antibodies in HNSCC; however, most human cancers show heterogeneous Trop2 expression, rendering this conclusion uncertain. Following on this work, in this report we use a panel of patient-derived xenograft models (PDX) and their cellular derivatives (conditionally reprogrammed cells, or CRCs^38^) to show that ErbB3 is an essential component of the tumor growth machinery in HNSCC. This essential function stems from a fundamental role for ErbB3 in HNSCC tumorigenesis, namely, an unexpected requirement for neuregulin and ErbB3 for maximal EGFR signaling. We also elucidate a novel therapeutic strategy involving co-inhibition of ErbB3 and its inhibitor, Trop2.

## Results

### HNSCC patient-derived xenografts are broadly sensitive to anti-ErbB3 antibody treatment

**Fig. 1:**
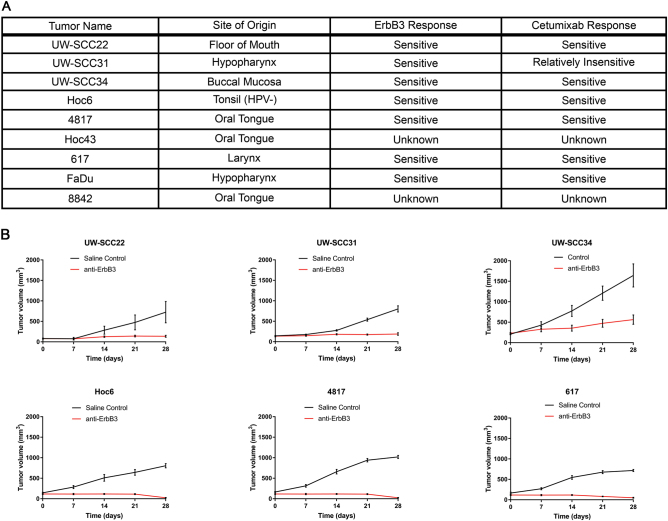
Description of patient-derived xenografts and their sensitivity to anti-ErbB3 antibody **a** The table shows the derivation of the models used in the study. **b** Growth curves of patient-derived xenografts treated with five weeks of anti-ErbB3 antibody. Tumors were allowed to grow to 150 mm^3^ prior to treatment for five weeks. Forty-eight hours after the 5th week residual tumor nodules were harvested. *n* = 10 mice per group. Differences in growth between treated and untreated mice were significant to *P* < 0.0001 (ANOVA) in all experiments

**Fig. 2:**
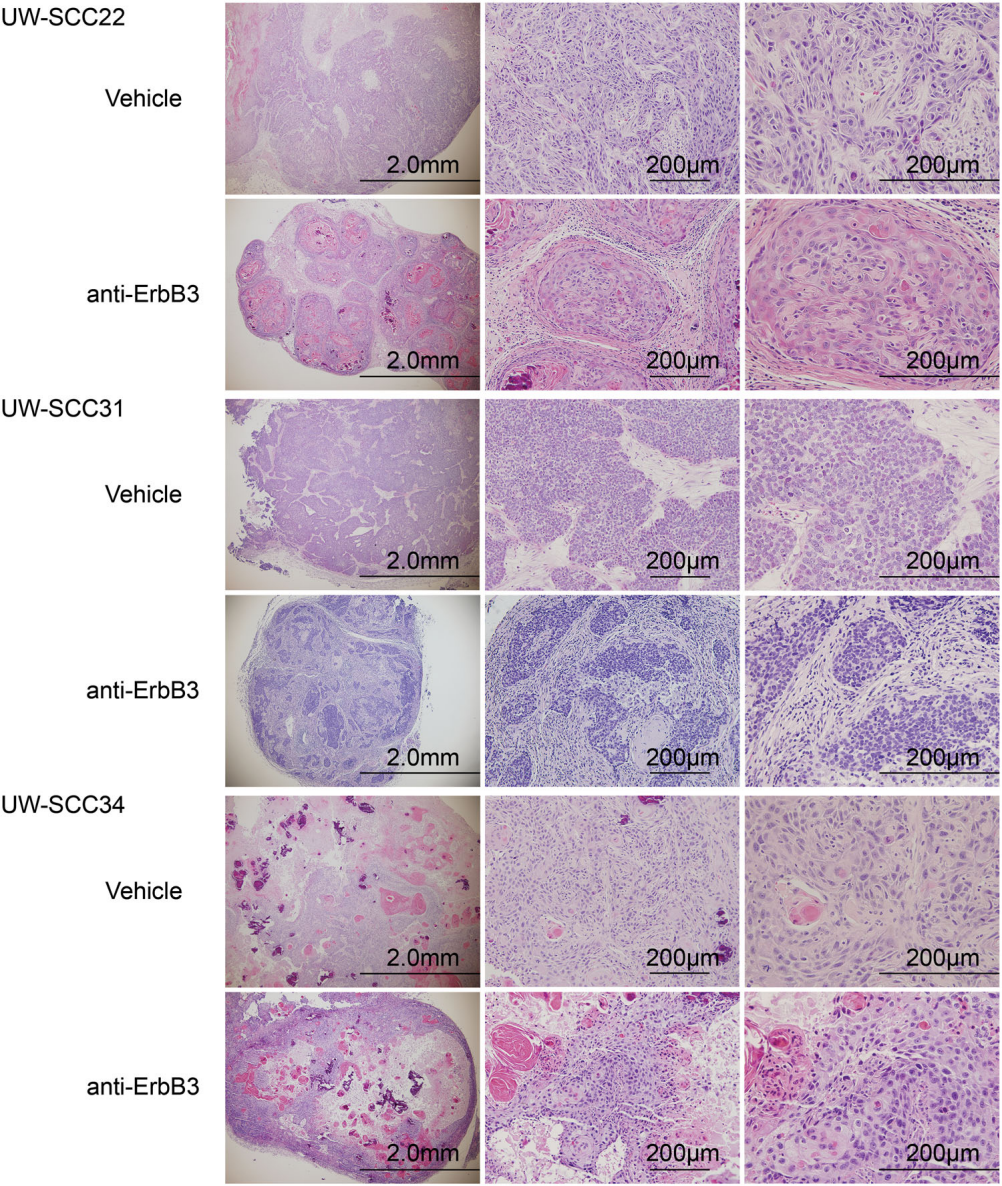
Assessment of residual nodules remaining after ErbB3 antibody treatment of mice harboring PDX tumors Hematoxylin and eosin stained tumor sections show varying degrees of cellularity in residual tumor nodules post-anti-ErbB3 antibody treatment. Shown are representative examples of tumor nodules harvested from mice bearing UW-SCC22, UW-SCC31, and UW-SCC34. In some nodules, very few or no cells were identified (UW-SCC31), whereas the degree of cellularity varied dramatically in other nodules

HNSCC is widely considered to be an EGFR-driven disease as its overexpression confers a poor prognosis^39^, Cetuximab is active in this disease, and most cell lines and many PDX models are sensitive to the growth inhibitory properties of cetuximab^40^. Recently, high protein levels of the kinase-dead family member ErbB3 and its ligand neuregulin have been shown to correlate with a poor prognosis in HNSCC^41^. In prior work, we identified an inhibitor of ErbB3 activation, the transmembrane protein, Trop2^29^ and showed that Trop2 depletion in HNSCC cell line xenografts predicted hypersensitivity to antibody-mediated ErbB3 inhibition. These data suggested that tumors with low Trop2 levels would be optimal targets for anti-ErbB3 antibodies. To further test this hypothesis, we obtained established^40^ as well as developed additional novel PDX models of HPV-negative HNSCC from multiple tumor subsites including oral cavity, oropharynx, hypopharynx, and larynx (Fig. 1a). We previously observed that a subset of HNSCC tumors exhibit very low Trop2 expression, but most HNSCC tumors show heterogeneous expression at the cellular level when assessed by immunohistochemistry^42^. Our panel of PDX models also showed heterogeneous Trop2 staining, which mimicked most primary HNSCC tumors that we have examined (Supplementary Fig. 1 and see Zhang et al.^42^) Using these models, we carried out a series of ErbB3 antibody treatment experiments. The six PDX models were permitted to grow to approximately 150 mm^3^ in nude mice (*n* = 10 per cohort) and then treated with an anti-ErbB3 antibody (Genentech) intraperitoneally using our established dosing schedule^42^. All six models showed a significant tumor response to treatment (*p* < 0.0001 for all). The tumors in three models (Hoc6, 4817 and 617) were consistently eradicated after treatment while in three other models (UW-SCC22, UW-SCC31, UW-SCC34), growth arrest was achieved (Fig. 1b). Histologic assessment of residual tumors demonstrated persistence of viable cells (Fig. 2). To confirm the activity was not due to a non-specific effect, mice bearing Hoc6 tumors (*n* = 4 per group) were treated with a second anti-ErbB3 antibody, mm-121 (Merrimack). Again, a significant anti-tumor effect was seen (Supplementary Fig. 2). While the activity of Cetuximab is complex, and is well established to involve both signaling and immunologic mechanisms, neither of the ErbB3 antibodies possess immunologic activity^19,26^ suggesting that antibody-mediated ErbB3 inhibition was sufficient to suppress growth of HNSCC PDXs established from multiple anatomic subsites.

### EGFR requires ErbB3 for maximal activation in HNSCC PDX and CRC models

**Fig. 3:**
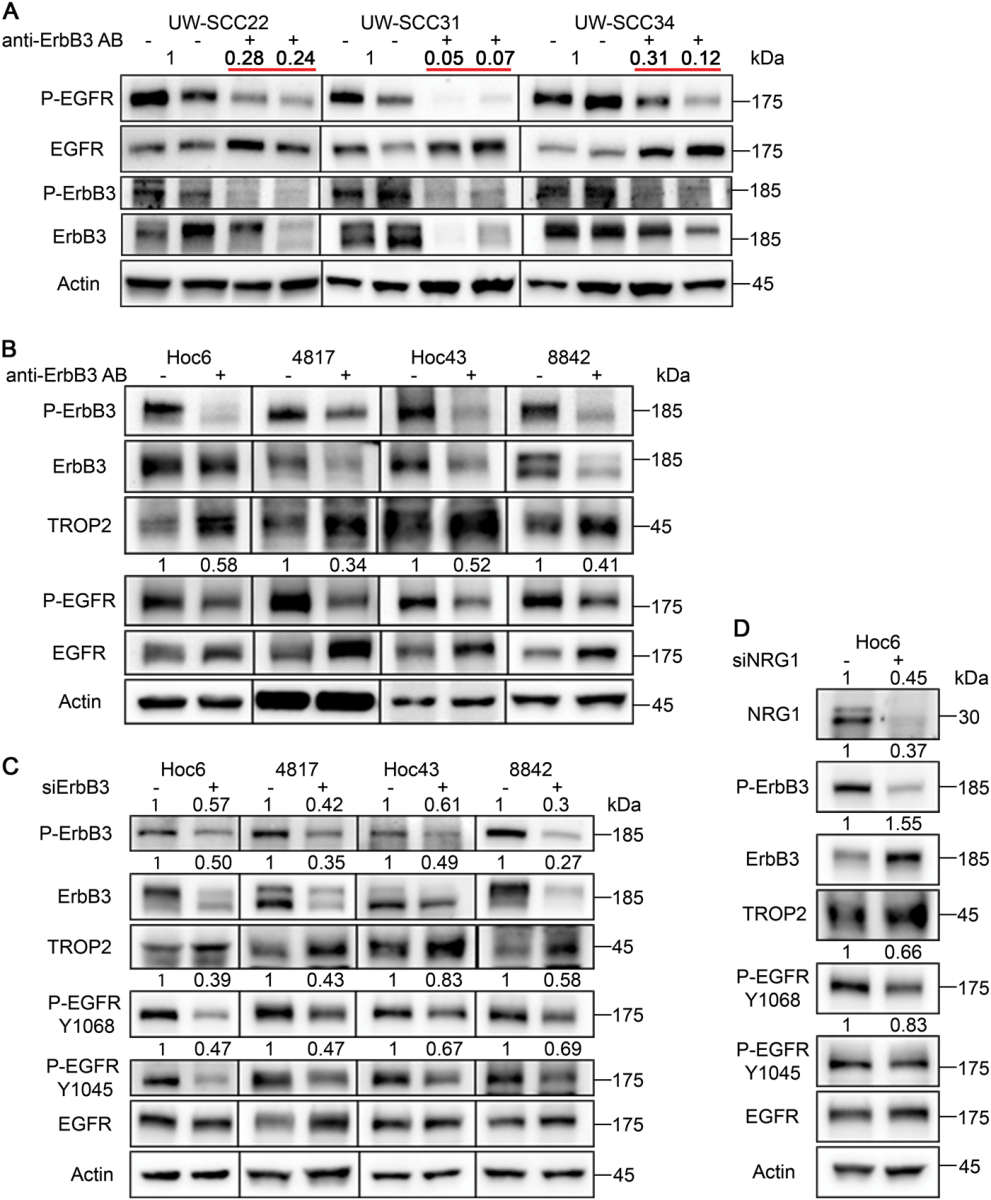
Requirement for ErbB3 for optimal EGFR activation in vivo and vitro **a** Tumor cell lysates obtained from tumor nodules containing persister cells after 5 weeks of treatment of anti-ErbB3 antibody treatment shows reduced levels of p-EGFR (Y1068) in ErbB3-antibody-treated mice compared to vehicle treatment. **b** Representative immunoblots of protein lysates from conditionally reprogrammed cells (CRCs) from four PDX models show reduced levels of p-EGFR after six days of incubation with anti-ErbB3 antibody (*n* = 3) or **c** three days after transient siRNA-mediated knockdown of ErbB3 (*n* = 3) or **d** neuregulin-1 (*n* = 3). Antibodies to both Y1068 and Y1045 were used. Relative decreases in phosphoprotein levels in control vs. experimental groups were quantified by photodensitometry after normalization to total EGFR or ErbB3. Squamous cells were separated from Swiss 3T3 feeder cells by differential trypsanization prior to preparation of protein lysates

We determined whether ErbB3 antibody treatment would influence levels of EGFR activation due to the generally accepted finding that HNSCC is an EGFR-driven disease. We harvested cells from residual tumor nodules from mice treated with ErbB3 antibody for five weeks and measured levels of activated EGFR in residual tumor cells (i.e., persister cells). Levels of activated EGFR measured by phospho-specific antibodies declined both in absolute terms and relative to total EGFR (Fig. 3a). In some tumor samples, total EGFR was observed to be increased after anti-ErbB3 antibody treatment (Fig. 3a). We confirmed that EGFR protein is only being measured in the tumor, as immunohistochemistry using an EGFR antibody cross-reactive to mouse and human in both untreated and ErbB3-antibody-treated tumors showed that only the HNSCC tumor cells and not the stroma express EGFR (Supplementary Fig. 3). Next, we sought to determine whether we could detect an effect of ErbB3 inhibition on EGFR activity in vitro, using patient-derived tumors as opposed to established cell lines. We adapted a culture system referred to as CRCs that has been harnessed to study targeted therapy sensitivity of primary tumor cells^43^. In this system, primary tumor cells are passaged on a layer of irradiated Swiss NIH/3T3 cells and harvested with differential trypsinization^38^. When CRCs from two patient-derived xenografts and two CRC models grown directly from a biopsy of the oral tongue were exposed to the anti-ErbB3 antibody for six days, we observed a similar decrease in EGFR activity and increased expression of Trop2 (Fig. 3b). Investigation into the unpredicted possibility that ErbB3 controls EGFR activation was further undertaken by the use of siRNAs against ErbB3^44^. Ninety-six hours after transfection of ErbB3 SMARTpool siRNAs, tumor cells from CRC cultures were harvested and again probed for levels of EGFR activation, experiments which showed elevated levels of Trop2 and reduced levels of p-EGFR even when ErbB3 was incompletely suppressed (Fig. 3c). To further demonstrate a causal relationship between ErbB3 and EGFR activation, we also transiently knocked down neuregulin-1, the ErbB3 ligand, and again examined levels of EGFR activation. In addition to suppressing ErbB3 activation and elevating Trop2 expression, incomplete neuregulin-1 depletion significantly reduced EGFR activation, consistent with the observed effects of the ErbB3 knockdown and ErbB3 antibody treatment (Fig. 3d). These Trop2 results are consistent with our previously published results finding Trop2 expression to be inversely proportional to ErbB3. We also investigated the effect of ErbB3 inhibition on ErbB2. Basal levels of ErbB2 were found to be very low by both western blotting and immunohistochemistry (Supplementary Figs. 4 and 5). Moreover, we did not observe consistent changes in p-ErbB2 levels with ErbB3 inhibition (Supplementary Figs. F4 and 5) suggesting that ErbB3 is not as tightly linked to ErbB2 function as it is to EGFR. Collectively, these data show for the first time that a neuregulin-ErbB3 axis controls EGFR activation in HNSCC CRCs and PDX models.

### ErbB3 regulates HIF1α in HNSCC CRCs

**Fig. 4:**
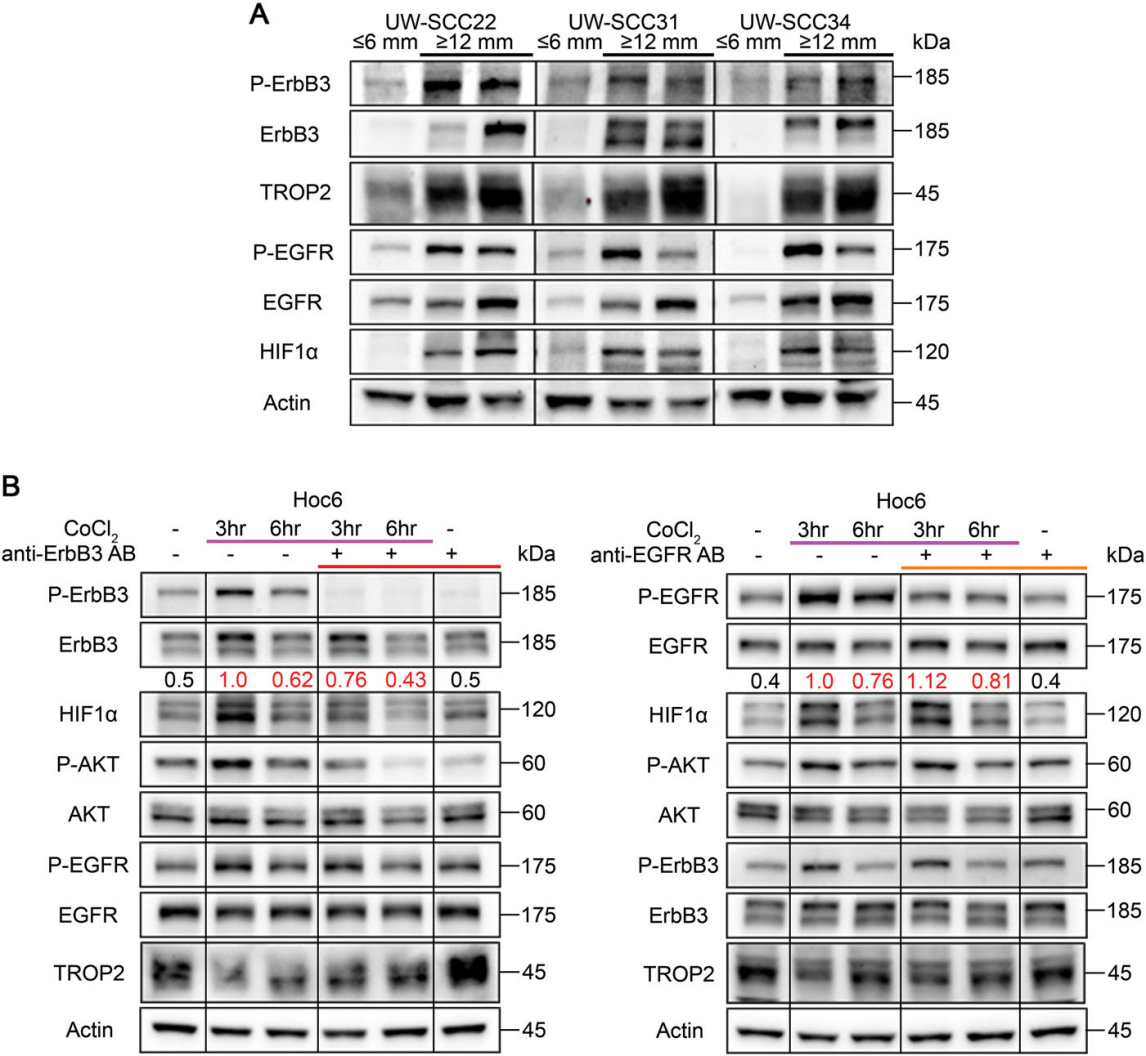
ErbB3 is essential for increased HIF1α stabilization in HNSCC **a** Protein lysates taken from larger ( ≥ 12 mm, *n* = 10) compared to smaller ( ≤ 6 mm, *n* = 3) tumors show that EGFR and ErbB3 are co-upregulated coincident with HIF1α stabilization in larger tumors. **b** Protein lysates from conditionally reprogrammed cells (Hoc6) exposed to CoCl_2_ show that two-hour pre-treatment with anti-ErbB3 antibody (left panel, *n* = 3) but not Cetuximab (right panel, *n* = 3) suppresses cobalt chloride-dependent HIF1α stabilization, which is maximal at three hours. Relative HIF1α levels in control vs. treatment groups were quantified by photodensitometry after normalization to actin

When performing the ErbB3 loss of function experiments in vitro, no decrease in cell number or proliferation was observed in anti-ErbB3-treated cells when compared to control cells (Supplementary Fig. 6A, B). This lack of efficacy in vitro is similar to that observed with PI3 kinase inhibition in other models and contexts (see Costa et al.^45^ and references therein). This suggested that ErbB3 signaling is particularly important for aspects of tumor growth related to the in vivo tumor microenvironment that are not required for in vitro growth. In three-dimensional in vitro culture systems, ErbB3 has been shown to be upregulated by HIF1α^46^, which is particularly important as tumors grow beyond their blood supply and encounter a hypoxic environment. In addition, in some contexts, EGFR promotes HIF1α stabilization primarily through its ability to activate the PI3 kinase-AKT pathway (Heeg et al. and references therein^47^). Therefore, we considered that the potent anti-tumor effect exerted by the ErbB3 antibody observed in vivo may also be related to a specific requirement for ErbB3 in tumor growth under hypoxic conditions. To test this possibility, we harvested PDX tumors from three of the models when they were either relatively small (≤6 mm in greatest diameter) or large (≥12 mm in greatest diameter), and measured ErbB3 activation and HIF1α levels, in addition to activated EGFR. Notably, ErbB3 and EGFR showed synchronously increased activation coincident with HIF1α stabilization in larger tumors (Fig. 4a. and Supplementary Fig. 7). To more fully investigate the relationship between ErbB3 in the HIF1α in the HOC6 CRC cultures, we used cobalt chloride (CoCl_2_) to mimic the hypoxic environment and promote HIF1α stabilization. Short-term exposure to this salt increased HIF1α and a panel of other hypoxia-inducible genes (Supplementary Fig. 8). When cells were pre-treated with the anti-ErbB3 antibody for two hours prior to the addition of CoCl_2_, the accumulation of HIF1α was significantly attenuated (Fig. 4b). Notably, Cetuximab treatment did not reduce HIF1α levels, but actually increased its stability (Fig. 4b, right panel). These results suggest that ErbB3 is required not only for optimal EGFR activity, but is essential for acute upregulation of HIF1α. Altogether, they explain the efficacy of ErbB3 inhibition in the PDX models.

### Upregulation of Trop2 in ErbB3-treated persister cells results in ErbB3-Trop2 synergistic targeting

**Fig. 5:**
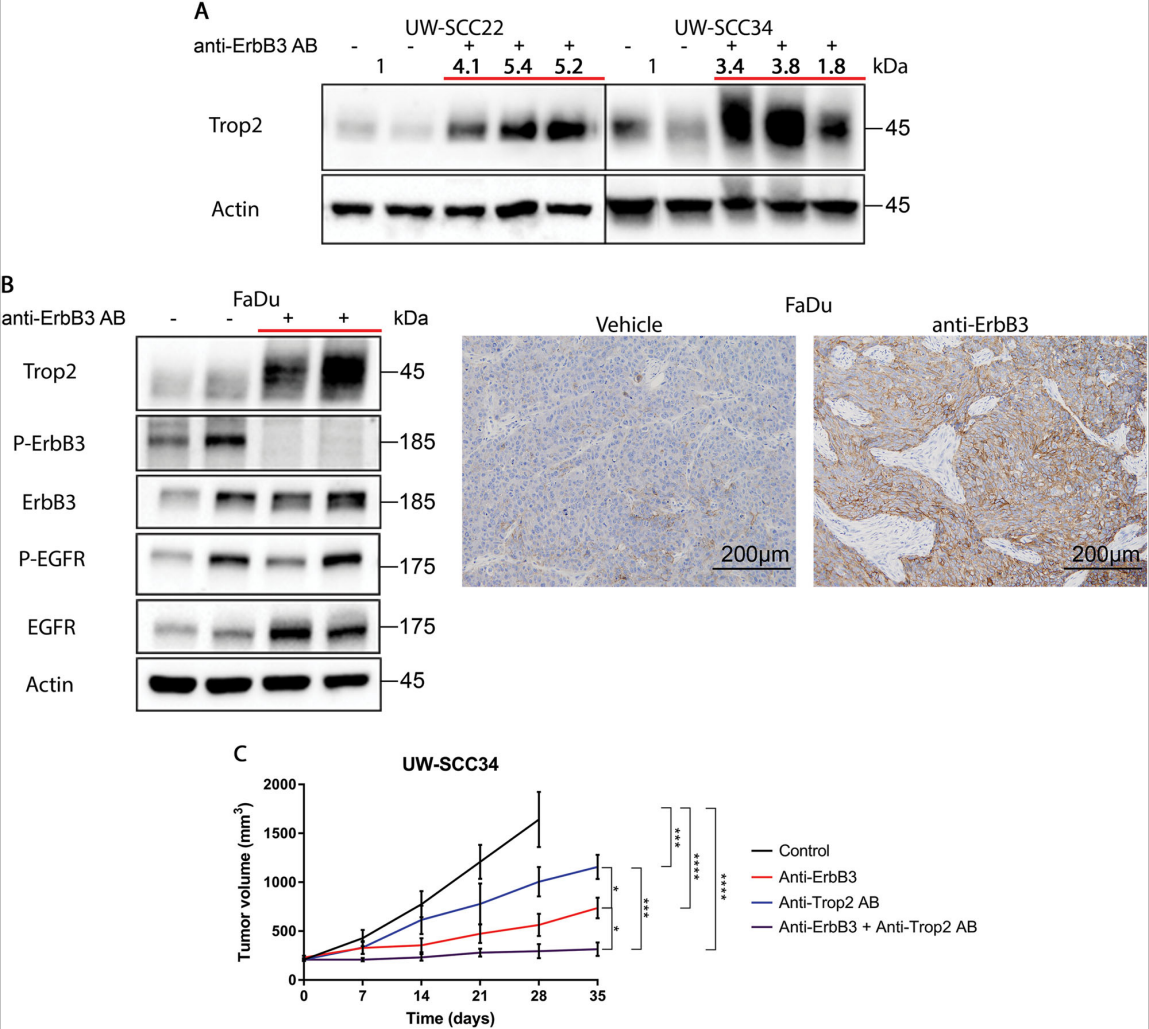
ErbB3 antibody treatment results in increased Trop2 levels **a** Protein lysates from tumor nodules harvested from mice after 5 weeks of anti-ErbB3 antibody treatment reveals increased levels of Trop2 in persister cells. **b** Right panels are photomicrographs of Trop2 staining in FaDu tumor xenografts after five weeks of anti-ErbB3 antibody treatment; left panels show immunoblots from the same tumor xenografts. *N* = 5 per group. Trop2 levels were quantified by photodensitometry and normalized to actin. **c** Synergistic activity of ant-Trop2 and anti-ErbB3 antibodies against UW-SCC34 xenografts growing in mice. *N* = 5 per group

As we previously reported, Trop2 binds to neuregulin-1, inhibiting its cleavage, and suppressing ErbB3 activation^42^. This observation explained the hypersensitivity to ErbB3 antibodies caused by RNAi-mediated depletion of Trop2 in HNSCC cell line xenografts that otherwise show very low levels of ErbB3 activation. As such, when we initiated ErbB3 treatment experiments in the PDX models, we expected to segregate ErbB3 sensitivity based on high vs. low Trop2 expression. Instead, we found that these models recapitulate the heterogeneity of Trop2 expression exhibited by most HNSCC human tumors (see Zhang et al.^42^ and Supplementary Fig. 1) and they also show low levels of ErbB3 expression by IHC (Supplementary Fig. 9). The data uncovered here suggests a more complex biological relationship between ErbB3 and Trop2. To gain a deeper understanding of this relationship, we asked whether ErbB3 antibody treatment would influence Trop2 expression. We started by examining the effects of ErbB3 antibody treatment on Trop2 expression levels in the ErbB3-persister cells that we were able to harvest post-ErbB3 treatment. In tumor material successfully obtained from ErbB3-treated persister cells from two PDX models, resistance to ErbB3 treatment was associated with an increase in Trop2 protein expression (Fig. 5a). We also examined the impact of ErbB3 treatment on Trop2 in a Trop2 low-expressing HNSCC cell line, FaDu. The tumor cells were implanted in mice (*n* = 5 tumors), allowed to grow to 150 mm^3^ and treated with the antibody to ErbB3. After three weeks, residual tumors were harvested and prepared for immunohistochemical and protein analysis. ErbB3 treatment resulted in increased Trop2 protein levels (Fig. 5b). These data led us to consider that ErbB3 antibody treatment may sensitize to treatment with anti-Trop2 therapeutics, which have now entering clinical trials. When the Trop2 antibody (Pfizer) was administered intraperitoneally on a weekly schedule to UW-SCC34 tumors, growth arrest comparable to single ErbB3 antibody treatment was observed. When the two antibodies were used simultaneously, they produced an anti-tumor effect that was more pronounced and synergistic compared to the observed single-agent activity of either antibody alone (Fig. 5c). These data indicate that co-inhibition of ErbB3 and Trop2 creates a synthetic dependency of each molecule on the other one resulting in a significant therapeutic benefit from combination treatment.

## Discussion

In this study, we investigated the requirement for ErbB3 in HNSCC. Targeting ErbB3 alone in a panel of PDXs revealed unexpected single-agent activity. The result was unexpected because numerous studies in HNSCC and other cancer cell lines generally indicated that co-targeting ErbB3 with EGFR or other pathways, chemotherapy, or radiotherapy, was required to yield a significant benefit from ErbB3 inhibition^26,28–32^. In addition, ErbB3 is frequently upregulated by various treatment strategies and as a result has more commonly been implicated as a mediator of resistance to therapy rather than an essential driver of tumorigenesis. Given the EGFR overexpression in HNSCC, this disease is considered to be an EGFR-driven tumor. Notably, ErbB3 is neither mutated, amplified, nor significantly overexpressed in HNSCC. Our data suggest, however, that ErbB3 is an essential component of the tumor growth machinery in HNSCC and the molecular details provided in this report offer some insight into why this is the case.

EGFR is capable of autophosphorylation, and as such, does not canonically require dimerization with ErbB2 or ErbB3^48–50^. In contrast, ErbB3 has been considered to be kinase-dead (although recent evidence may eventually overturn this idea^50^, and, therefore, requires a dimerization partner. Either EGFR or ErbB2 is considered essential for the ability of ErbB3 to activate downstream signaling. While neuregulin stimulation has been shown to result in EGFR transactivation in cultured cells^51^, neither neuregulin nor ErbB3 have ever been shown to be essential for the activity of EGFR. We observed that ErbB3-inhibited patient-derived tumors and derivative cultured CRCs are unable to maintain maximal EGFR signaling. This diminution occurred whether ErbB3 activity was decreased by ErbB3 antibody treatment, or siRNA to ErbB3 or neuregulin-1. Altogether, the data suggest that in HNSCC, full EGFR activation is dependent on the ErbB3-neuregulin-1 axis, a hierarchy that has not been previously demonstrated in any tumor type. These data are also consistent with and provide a reason for the observation that compared to other cancers, HNSCC tumors have among the highest levels of neuregulin-1. These levels increase in recurrent tumors that arise after curative treatments with radiation and chemotherapy^52^.

Another key finding identified in this report explains a feature of PI3kinase signaling inhibition that has been observed in several tumor models but not well understood, namely the lack of observed efficacy of ErbB3 inhibition in vitro relative to the profound anti-tumor effect in vivo. Other groups have reported a similar discordance with inhibitors of the PI3 kinase pathway^45^, but this observation has not been explained experimentally. This lack of in vitro efficacy with concomitant in vivo activity led us to consider a role for ErbB3 in responding to the tumor microenvironment. In support of this idea, we noted that as the PDXs grew in size they exhibited a dramatic increase in total and activated EGFR, ErbB3, and the hypoxia regulated transcription factor, HIF1α. EGFR itself has been reported to drive HIF1α expression in some systems^53^, but we did not observe any effect of Cetuximab on HIF1α levels in controlled CoCl_2_ experiments. Interestingly, 3D tissue culture assays of HNSCC cells have shown that linked HIF1α upregulates ErbB3^46^. Our data suggest that regulation may occur in both directions, as suppression of ErbB3 significantly reduced the ability of CoCl_2_ -induced hypoxia signaling to upregulate HIF1α. Collectively, these data point to ErbB3 as a mediator of signaling required for survival in the hostile tumor microenvironment, a requirement that is a likely source of the anti-tumor activity of anti-ErbB3 antibodies.

The lack of high-frequency actionable mutations in HNSCC presents a barrier to the development of novel targeted treatment approaches, particularly in smoking-related disease which is a difficult-to-treat group of tumors with a high mortality rate. Despite extensive genomic investigation, genetic alterations of molecular drivers such as EGFR (other than PI3 kinase) have not been identified in HNSCC^4,5,11^, and the lack of mutations in HNSCC corresponds with the failure to find genetic addictions in this disease. The data presented here in this report not only underscore the limitations of therapeutic inhibition of non-genetically altered targets, but also suggest that strategies to overcome these limitations are within reach in some cases. While ErbB3 inhibition alone exerts potent anti-tumor activity, some “persister” cells are able to survive and show upregulation of Trop2, the negative regulator of ErbB3^42^. The ability of dual targeting of ErbB3 and Trop2 to more completely suppress tumorigenesis suggests that ErB3 treatment creates a novel dependency on Trop2. Therefore, the ErbB3-Trop2 interaction uncovered here provides a treatment possibility that is predicted to be highly effective and a model for inhibiting other non-mutated (e.g., non-addicted) targets. The extent to which dual inhibition will provoke a potent addiction in patients should be tested formally in clinical trials.

## Materials and methods

### Cell line maintenance—Hoc6, Hoc43, 4817, 8842

Swiss 3T3 fibroblasts, aka feeder cells, at lower passage number were trypsinized and collected as a cell pellet. The cell pellet was irradiated at 30 Gray (3000 rad). After irradiation, feeder cells were plated at 70–90% density in complete Dulbecco's Modified Eagle Medium (DMEM) media. Irradiated feeder cells were allowed to attach at least three hours prior to plating CRC lines over feeder cells in F media. F media: Complete DMEM 1x (DMEM—Gibco # 11965-092; 10% FBS—Gibco #16140-071; 1% l-glutamine—Gibco # 25030-081; 1% Pen/Strep—Gibco # 15140-122); F-12 Nutrient Mix 1x—Gibco #11765-054; 25 microgram/milliliter (µg/ml) Hydrocortisone—Sigma H-0888; 0.125 µg/ml EGF—Invitrogen #PHG0311L; 5 milligram/milliliter (mg/ml) Insulin—Sigma #I-5500; 11.7 microMolar (µM) cholera toxin—Sigma #C-8052. Cells were grown more the 70% dense then split with differential trypsinization. Co-cultures were briefly washed with sterile phosphate-buffered saline (PBS) and incubated with 0.05% trypsin solution at room temperature for 1–3 min (min). Flasks were gently tapped until all feeders detached from substrate as confirmed with close monitoring by phase microscopy. Detached cells were removed by aspiration. Tightly adherent epithelial cells were rinsed again with PBS, and then re-trypsinized with 0.25% at 37 °C for 3–5 min. Cells were dispersed into single suspension with gentle pipetting, pelleted and resuspended in F medium for passaging. Cell line FaDu was maintained in DMEM/F12 media. DMEM/F12 media: DMEM/F12—Gibco #11330–032; 10% FBS—Gibco #16140-071; 25 µg/ml Hydrocortisone—Sigma H-0888; 1% Pen/Strep—Gibco # 15140-122.

### Western blots analysis

For CRC cell line protein expression, Swiss 3T3 fibroblasts were first trypsinized off with 0.05% trypsin. Culture plates were washed with buffer and cell pellets were collected with cell scrapers and lysed with standard RIPA buffer supplemented with 1 milliMolar (mM) sodium orthovanidate, 1 mM phenylmethylsulfonyl fluoride, and protease inhibitor cocktail P8340 (Sigma-Aldrich). Cell pellets were incubated on ice for twenty min with constant agitation, centrifuged for two min and supernatants were collected. For xenograft tumor protein expression, tumor chunks were homogenized using pestles and lysed in RIPA buffer for 20 min before sonication, centrifugation for 10 min at 4 °C, and supernatant collection. Total protein concentrations were determined by Quick Start™ Bradford Assay (BioRad).

Forty microgram of protein lysate were added per sample, diluted in millipure H_2_O to 20 microliters (µl), and diluted further with 5x sodium dodecyl sulfate polyacrylamide gel electrophoresis (SDS-PAGE) Loading Buffer. Samples with boiled at 95 °C for 5 min and ran through an SDS-PAGE gel. Samples were electrotransfered onto 0.2 µM polyvinylidene fluoride membranes. Membranes were blocked with 5% milk in 1x PBS-0.1% Tween20 for 30 min at room temperature. Primary antibodies diluted in PBS-0.1% Tween20, 3% bovine serum albumen, 0.02% sodium azide (NaN_3_) solution were incubated overnight on a rocker at 4 °C. After primary antibody incubation, membranes were washed three times in 1x PBS-0.1% Tween20 for 8 min each. Species-specific HRP conjugated secondary monoclonal antibodies were diluted in 5% milk in 1x PBS-0.1% Tween20 at a concentration of 1:5000 and incubated for 1 h at room temperature. After secondary antibody incubation, membranes were washed three times in 1x PBS-0.1% Tween20 for 8 min each. SuperSignal West Femto Maximum Sensitivity Substrate (Thermo) was used for visualization. Images were acquired using Chemidoc XRS+ imaging system and Image Lab Software. Primary antibodies used are as follows: anti-Phospho-EGFR (Tyr1068) Cell Signaling Technology #3777, anti-Phospho-EGFR (Tyr1045) Cell Signaling Technology #2237, anti-EGFR Cell Signaling Technology #4267, anti-Phospho-ErbB3 (Tyr1289) Cell Signaling Technology #4791, anti-ErbB3 Cell Signaling Technology #12708, anti-NRG1 R & D Systems AF-296-NA, anti-Actin Sigma-Aldrich A1978, anti-Tubulin Sigma-Aldrich T6199, anti-HIF1α Abcam ab51608, and anti-Trop2 R & D Systems AF650.

### Anti-ErbB3 treatment in vitro with Dl3.6b and mm121

Irradiated Swiss 3T3 fibroblasts were plated in 60 millimeter (mm) plates at 500,000 cells in 2 ml media. CRC lines were plated on irradiated fibroblasts 3–5 h later at 50,000 cells per well in CRC culture media. Twenty-four hours following CRC culture splitting procedure, regular F media (control samples) and Dl3.6b (100 µg/ml) or mm-121 (170 µg/ml) supplemented F media (experimental samples) was added. Following incubation at 37 °C for 72 h, fresh F media with and without anti-ErbB3 antibody Dl3.6b (Genentec) or mm-121 (Merrimack) was added. Samples were incubated for another 72 h. Samples were washed with PBS and Swiss 3T3 fibroblasts were detached with 0.05% Trypsin. Culture plates were washed with PBS and cell pellets were collected with cell scrapers and lysed with RIPA buffer.

### Cell counting

Irradiated Swiss 3T3 fibroblasts were plated in six-well plates at 280,000 cells in 2 ml media. CRC lines were plated on irradiated fibroblasts 3–5 h later at 50,000 cells per well in CRC culture media. Twenty-four hour following CRC culture splitting procedure, regular F media (control samples) or Dl3.6b (100 µg/ml) supplemented F media (experimental samples) was added. Following incubation at 37 °C for 72 h, fresh F media with and without Dl3.6b was added. Samples were incubated for another 72 h. Samples were washed with PBS and Swiss 3T3 fibroblasts were detached with 0.05% Trypsin/EDTA. CRC lines were detached with 0.25% Trypsin. Aliquots were diluted 1:2 with Trypan Blue and a viable cell count was obtained using a hemocytometer.

### CoCl_2_

Irradiated Swiss 3T3 fibroblasts were plated in 60 mm plates at 500,000 cells in 2 ml media. CRC lines were plated on irradiated fibroblasts 3–5 h later at 50,000 cells per well in CRC culture media. Cells were grown to 50% confluence, and then either anti-ErbB3 Dl3.6b (100 µg/ml) or anti-EGFR Cetuximab (14 µg/ml) was added. CoCl_2_ was added for a final concentration of 200 µM to antibody-treated and control plates 2 h later. Samples were collected following either three or six hours incubation. Irradiated fibroblasts were trypsinized off, culture plates were washed with buffer, and cell pellets were collected with cell scrapers and lysed with RIPA buffer.

### siRNA-mediated ErbB3 and NRG1 knockdowns

CRC cells (Hoc6, 4817, Hoc43, 8842) were transiently transfected with ErbB3 siRNA (siErbB3; ON-TARGETplus, SMARTpool # L-003127; Dharmacon), siNRG1 (ON-TARGETplus, SMARTpool # L-004608; Dharmacon), or non-targeting siRNA (siNT; ON-TARGETplus Non-targeting Pool, #D-001810; Dharmacon) using Lipofectamine 2000 according to the manufacturer’s instructions (Life Technologies).

### Xenograft studies

Mouse xenograft experimental protocols were approved by the Institutional Animal Care and Use Committee (IACUC) at Washington University in St. Louis. Animals were maintained and evaluated under pathogen-free conditions following IACUC guidelines (St. Louis, MO). Athymic nude mice (4 to 6-weeks-old females) were obtained from Jackson Laboratories (Bar Harbor, ME). PDXs were surgically grafted in the flanks of each mouse utilizing sterile technique and follow up care. CRC tumor xenografts were generated by separating off Swiss 3T3 feeder cells with 0.05% trypsin and washing with Dulbecco’s PBS, followed by 0.25% trypsin to produce a suspension of CRC cells in media. In all, 2.0 × 10^6^ cells in media and 30% Matrigel (BD Biosciences) were injected subcutaneously into both flanks of each mouse. Tumors were measured in length and width with calipers several times per week and volumes were calculated using the formula (length X width^2^)/2. Tumors were allowed to reach an approximate volume of 150 mm^3^ before being round-robined into treatment groups. Mice were treated with intraperitoneal injections of anti-ErbB3 antibody (Genentech) at 25 milligram/kilogram (mg/kg) weekly, anti-Trop2 antibody (Pfizer) at 20 mg/kg weekly, the combination of the two at the same dose and schedule, or with PBS for negative control. Mice were sacrificed according to IACUC approved protocol upon reaching two centimeters diameter in one dimension or two days after last treatment. Tumor material was harvested for both protein analyses by western by snap freezing in liquid nitrogen and histological studies by formalin fixation.

### Histological studies

Tumor samples were fixed in ten percent neutral buffered formalin for 24–48 h and embedded in paraffin wax at the end of experiments. Sequential slices of each tumor were transferred to charged microscope slides and samples were processed for Hematoxylin and Eosin and protein expression (anti-Trop2 R & D Systems AF650 1:500). Samples were deparaffinized with two, five min washes in xylenes. Samples were then rehydrated through ethanol series. Heat-induced antigen retrieval was then performed using either citric acid solution (H3300) or basic EDTA solution (CTS013) according to primary antibody recommendation utilizing a microwave. Primary antibodies were diluted in antibody solution: PBS, 1% normal donkey serum, 0.3% Triton X-100, and NaN_3_ 0.0003% pH 7.4. Samples were incubated overnight with primary antibody at 4 °C in moist chamber. The following day, samples were washed three times with PBS for 5 min each and incubated with species-specific biotinylated secondary antibody for an hour at room temperature. Samples were again washed three times with PBS and then incubated with avidin-biotin HRP complex solution (Vector) prepared 30 min before use for 30 min. Following three, 5 min washes, samples were developed using diaminobenzidine substrate and counterstained with hematoxylin. Slides were then dehydrated through ethanol series and sealed using xylene based solution. Histology images were collected with Olympus BX51 microscope with Olympus DP70 camera using 4x, 20x, and 40x objective lenses.

## Electronic supplementary material

Supplemental Materials

Supplemental Figure 1

Supplemental Figure 2

Supplemental Figure 3

Supplemental Figure 4

Supplemental Figure 5

Supplemental Figure 6

Supplemental Figure 7

Supplemental Figure 8

Supplemental Figure 9
