# Supplementary material for: Anti-Trop2 blockade enhances the therapeutic efficacy of ErbB3 inhibition in head and neck squamous cell carcinoma
Source: Cell Death Dis. 2018 Jan 5;9(1):5. doi: 10.1038/s41419-017-0029-0 (PMC5849045; doi:10.1038/s41419-017-0029-0)
Supplement: Supplementary file 1 — Supplemental Materials [file 41419_2017_29_MOESM1_ESM.pdf]

Supplemental Materials and Methods

Xenograft studies

Mouse xenograft experimental protocols were approved by the Institutional Animal Care and Use Committee (IACUC) at Washington University in St. Louis. Animals were maintained and evaluated under pathogen-free conditions following IACUC guidelines (St. Louis, MO). Animals were maintained and evaluated under pathogen-free conditions following IACUC guidelines (St. Louis, MO). Athymic nude mice (4 to 6 weeks old females) were obtained from Jackson Laboratories (Bar Harbor, ME). Patient derived xenografts were surgically grafted in the flanks of each mouse utilizing sterile technique and follow up care. CRC tumor xenografts were generated by separating off Swiss 3T3 feeder cells with 0.05% trypsin and washing with Dulbecco's phosphate buffered saline, followed by 0.25% trypsin to produce a suspension of CRC cells in media. 2 X 10^6 cells in media and 30% Matrigel (BD Biosciences) were injected subcutaneously into both flanks of each mouse. Tumors were measured in length and width with calipers several times per week and volumes were calculated using the formula (length X width^2)/2. Tumors were allowed to reach an approximate volume of 150 mm^3 before being round-robined into treatment groups. Mice were treated with intraperitoneal injections of anti-ErbB3 antibody (Merrimack) at 25 mg/kg every three days, or with PBS for control. Mice were sacrificed according to IACUC approved protocol upon reaching two centimeters diameter in one dimension or two days after last treatment. Tumor material was harvested for both protein analyses by western by snap freezing in liquid nitrogen and histological studies by formalin fixation.

Histological Studies

Tumor samples were fixed in ten percent neutral buffered formalin for 24 to 48 hours and embedded in paraffin wax at the end of experiments or upon reaching two cm diameter in one direction. Sequential slices of each tumor were transferred to charged microscope slides and samples were processed for Hematoxylin & Eosin and protein expression (anti-EGFR Cell Signaling Technology #4267 1:50, anti-ErbB3 Cell Signaling Technology #12708 1:100, anti-ErbB2 Cell Signaling Technology #4290 1:200, HIF1α Abcam ab51608 1:100, and anti-Trop2 R & D Systems AF650 1:100). Samples were deparaffinized with two, five minute washes in xylenes. Samples were then rehydrated through ethanol series. Heat Induced Antigen Retrieval was then performed using either citric acid solution (H3300) or basic EDTA solution (CTS013) according to primary antibody recommendation utilizing a microwave. Primary antibodies were diluted in antibody solution: PBS, 1% Normal Donkey Serum, 0.3% Triton X-100, and NaN_3 0.0003% pH 7.4. Samples were incubated overnight with primary antibody at 4^0C in moist chamber. The following day, samples were washed three times with PBS for five minutes each and incubated with species specific biotinylated secondary antibody for an hour at room temperature. Samples were again washed three times with PBS and then incubated with avidin-biotin HRP complex solution (Vector) prepared thirty minutes before use for thirty minutes. Following three, five minute washes, samples were developed using diaminobenzidine substrate and counterstained with hematoxylin. Slides were then dehydrated through ethanol series and sealed using xylene based solution. Histology images were collected with Olympus BX51 microscope with Olympus DP70 camera using 4x, 20x, and 40x objective lenses.

Western Blots analysis

For CRC cell line protein expression, Swiss 3T3 fibroblasts were first trypsinized off with 0.05% trypsin. Culture plates were washed with buffer and cell pellets were collected with cell scrapers and lysed with standard RIPA buffer supplemented with 1 milliMolar (mM) sodium orthovanidate, 1 mM phenylmethylsulfonyl fluoride (PMSF), and protease inhibitor cocktail P8340 (Sigma-Aldrich). Cell pellets were incubated on ice for twenty min with constant agitation, centrifuged for two min and supernatants were collected. For xenograft tumor protein expression, tumor chunks were homogenized using pestles and lysed in RIPA buffer for twenty min before sonication, centrifugation for 10 min at 4°C, and supernatant collection. Total protein concentrations were determined by Quick Start™ Bradford Assay (BioRad).

40 µg of protein lysate were added per sample, diluted in millipure H_2O to 20 microliters (µl), and diluted further with 5x sodium dodecyl sulfate polyacrylamide gel electrophoresis (SDS-PAGE) Loading Buffer. Samples with boiled at 95°C for 5 min and ran through an SDS-PAGE gel. Samples were electrotransfered onto 0.2 µM Polyvinylidene fluoride (PVDF) membranes. Membranes were blocked with 5% milk in 1x PBS-0.1% Tween20 for 30 min at room temperature. Primary antibodies diluted in PBS-0.1% Tween20, 3% Bovine Serum Albumen (BSA), 0.02% sodium azide ($NaN_{3)}$ solution were incubated overnight on a rocker at 4°C. After primary antibody incubation, membranes were washed three times in 1x PBS-0.1% Tween20 for 8 min each. Species-specific HRP conjugated secondary monoclonal antibodies were diluted in 5% milk in 1x PBS-0.1% Tween20 at a concentration of 1:5000 and incubated for 1 hour at room temperature. After secondary antibody incubation, membranes were washed three times in 1x PBS-0.1% Tween20 for 8 min each. SuperSignal West Femto Maximum Sensitivity Substrate (Thermo) was used for visualization. Images were acquired using Chemidoc XRS+ imaging

system and Image Lab Software. Primary antibodies used are as follows: anti-Phospho-ErbB2 Cell Signaling Technology #2243, anti-ErbB2 Cell Signaling Technology #4290, anti-Carbonic anhydrase IX Novus Biologicals NB100-417, Hypoxia Pathway Antibody Panel GeneTex GTX3000061, and anti-βActin Sigma-Aldrich A1978.

BRDU

Irradiated Swiss 3T3 fibroblasts were plated in 4 well chamber slides at 50,000 cells in 0.5 mL media. CRC lines were plated on irradiated fibroblasts 3-5 hours later at 10,000 cells per well in F media. 24 hours following CRC culture splitting procedure, regular F media (control samples) or Dl3.6b (100μg/mL) supplemented F media (experimental samples) was added. Following incubation at 37°C for 72 hours, fresh F media with and without Dl3.6b was added. Samples were incubated for another 72 hours. Samples were washed 3 times with PBS before each of the following steps: incubation at 37°C for 1 hour in BRDU labeling medium (1:1000), incubation at 4°C for 20 minutes in ethanol fixative, incubation at 37°C in mouse anti-BRDU antibody solution, incubation at 37°C in anti-mouse fluorescein conjugated solution, mounting with solution containing DAPI. Samples were cured in the dark for 24 hours before imaging. Images were collected with Olympus IX70 microscope with Olympus DP72 camera using 20x objective lens. CellSens Entry software was used for capturing images. Swiss 3T3 fibroblasts were excluded from counts visually.

$CoCl_2$

Irradiated Swiss 3T3 fibroblasts were plated in 60 mm plates at 500,000 cells in 2 ml media. CRC lines were plated on irradiated fibroblasts 3-5 hours later at 50000 cells per well in CRC culture media. Cells were grown to 50% confluence, and then $CoCl_2$ was added to a final concentration of 200μM. Samples

were collected following three hours incubation. Irradiated fibroblasts were trypsinized off, culture plates were washed with buffer, and cell pellets were collected with cell scrapers and lysed with RIPA buffer.

Supplemental Figures

Supplemental Figure 1. Heterogeneity of Trop2 expression is in HNSCC and PDX models. (A&B) Photomicrograph of Trop2 antibody stained section of a representative oral cavity tumor. (C-H) Photomicrographs of Trop2 stained tumor sections from the patient-derived xenograft models used in this study. Tumors were counterstained with hematoxlyin.

Supplemental Figure 2. Growth curve of PDX Hoc6 treated with the anti-ErbB3 antibody mm-121 (Merrimack Pharmaceuticals). Tumors were implanted into nude mice (n=5) and allowed to grow to 150 mm^3 prior to treatment for five weeks starting at time zero. Differences in growth between treated and untreated mice were significant to $P<0.0001$ (ANOVA).

Supplemental Figure 3. EGFR status of tumor and stroma of PDX models. Representative microscopy images of immunohistochemical staining of PDX tumor sections. Sections were counterstained with hematoxylin. EGFR staining shows specific staining in tumor cells but not stromal elements. Pre- and post-treatment sections are shown in microscopy fields of nodules with high levels of cellularity. All images are 20x and scale bars are 200µm.

Supplemental Figure 4. Western Blot Characterization of ErbB2 Across Conditions: (A) PDX control and post-anti-ErbB3 antibody treatment in Vivo, (B) in Vitro treatment of CRC cell lines with anti-ErbB3 antibody, (C) in Vitro treatment of CRC cell lines with siErbB3 SMARTpool, (D) in vitro treatment of Hoc6 CRC cell line with siNRG1 SMARTpool, (E) In

Vivo characterization of PDX models comparing small and large tumors, (F) in Vitro treatment of Hoc6 CRC cell line with $CoCl_2$ and anti-ErbB3 antibody, (G) in Vitro treatment of Hoc6 CRC cell line with $CoCl_2$ and anti-EGFR antibody, and (H) in Vivo treatment of FaDu cell line xenografts with anti-ErbB3 antibody.

Supplemental Figure 5. Immunohistochemistry for ErbB2 of PDX models. Representative microscopy images of immunohistochemical staining of PDX tumor sections for total ErbB2. Control and anti-ErbB3 treated sections are shown in microscopy fields of nodules with high levels of cellularity. Expression of ErbB2 is low and variable with no consistent pattern across conditions. All images are 20x and scale bars are 200μm.

Supplemental Figure 6. Lack of effect of anti-ErbB3 antibody treatment on cell viability and proliferation in vitro in conditionally reprogrammed cell cultures. (A) Conditionally reprogrammed cells were treated for six days with anti-ErbB3 antibody, separated from Swiss 3T3 feeder cells by differential trypsinization, and then were enumerated by Trypan blue assay (n=6). No statistical difference in cell viability was observed between the two groups (Fisher's exact test). (B) Conditionally reprogrammed cells were treated for six days with anti-ErbB3 antibody before cultures were labeled with bromodeoxyuridine (BRDU) and 4',6-diamidino-2-phenylindole (DAPI). Cultures were than imaged under a fluorescence microscope and the ratio of BRDU to DAPI positive cells was enumerated (n=5). No statistical effect of ErbB3 antibody treatment was observed in vitro. DAPI positive feeder cells were excluded from the count by visual inspection under light microscopy.

Supplemental Figure 7. HIF1α status of tumor and stroma of PDX models. Representative microscopy images of immunohistochemical staining of PDX tumor sections for Hif1α. Counterstained with hematoxylin. All images are 20x and scale bars are 200μm.

Supplemental Figure 8. Verification of induction of Hypoxia in Vitro. Western blot analysis of several hypoxia pathway proteins after 3 hours of $CoCl_2$ (n=3). Photodensitometry was performed using ImageLab software normalizing to actin and untreated control with average displayed above representative blot. Expression levels went up in 5/6 proteins tested indicating that hypoxic conditions were induced by $CoCl_2$ treatment.

Supplemental Figure 9. Immunohistochemistry for ErbB3 of PDX models. Representative microscopy images of immunohistochemical staining of PDX tumor sections. Control and post-treatment sections are shown in microscopy fields of nodules with high levels of cellularity. ErbB3 expression is low in all samples correlating with western blot analysis, which necessitates long exposures to view protein. All images are 20x and scale bars are 200μm.
