# Supplementary figures and images for: Anti-Trop2 blockade enhances the therapeutic efficacy of ErbB3 inhibition in head and neck squamous cell carcinoma
Source: Cell Death Dis. 2018 Jan 5;9(1):5. doi: 10.1038/s41419-017-0029-0 (PMC5849045; doi:10.1038/s41419-017-0029-0)

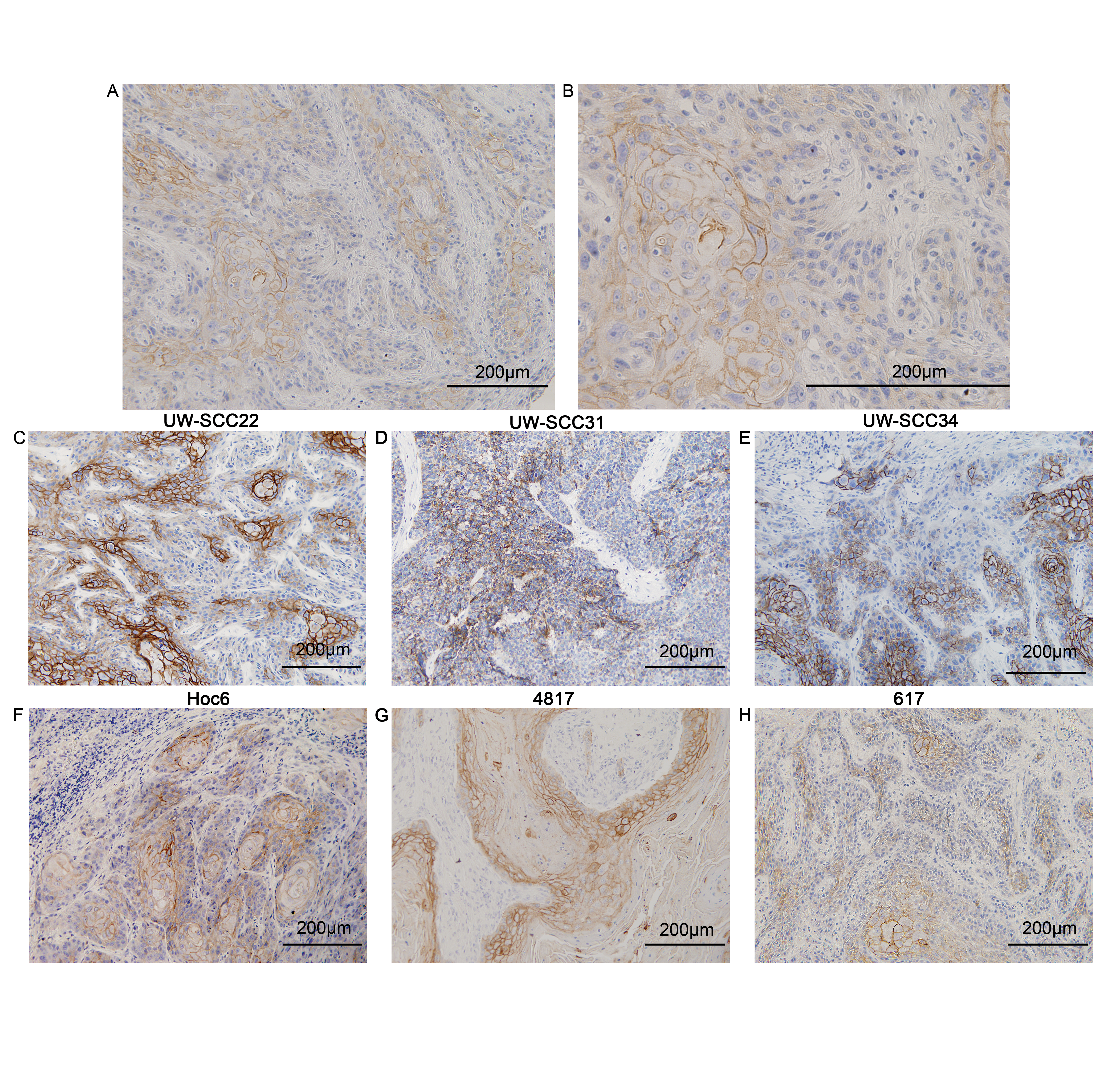

Supplement: Supplementary file 2 — Supplemental Figure 1 [file 41419_2017_29_MOESM2_ESM.png]

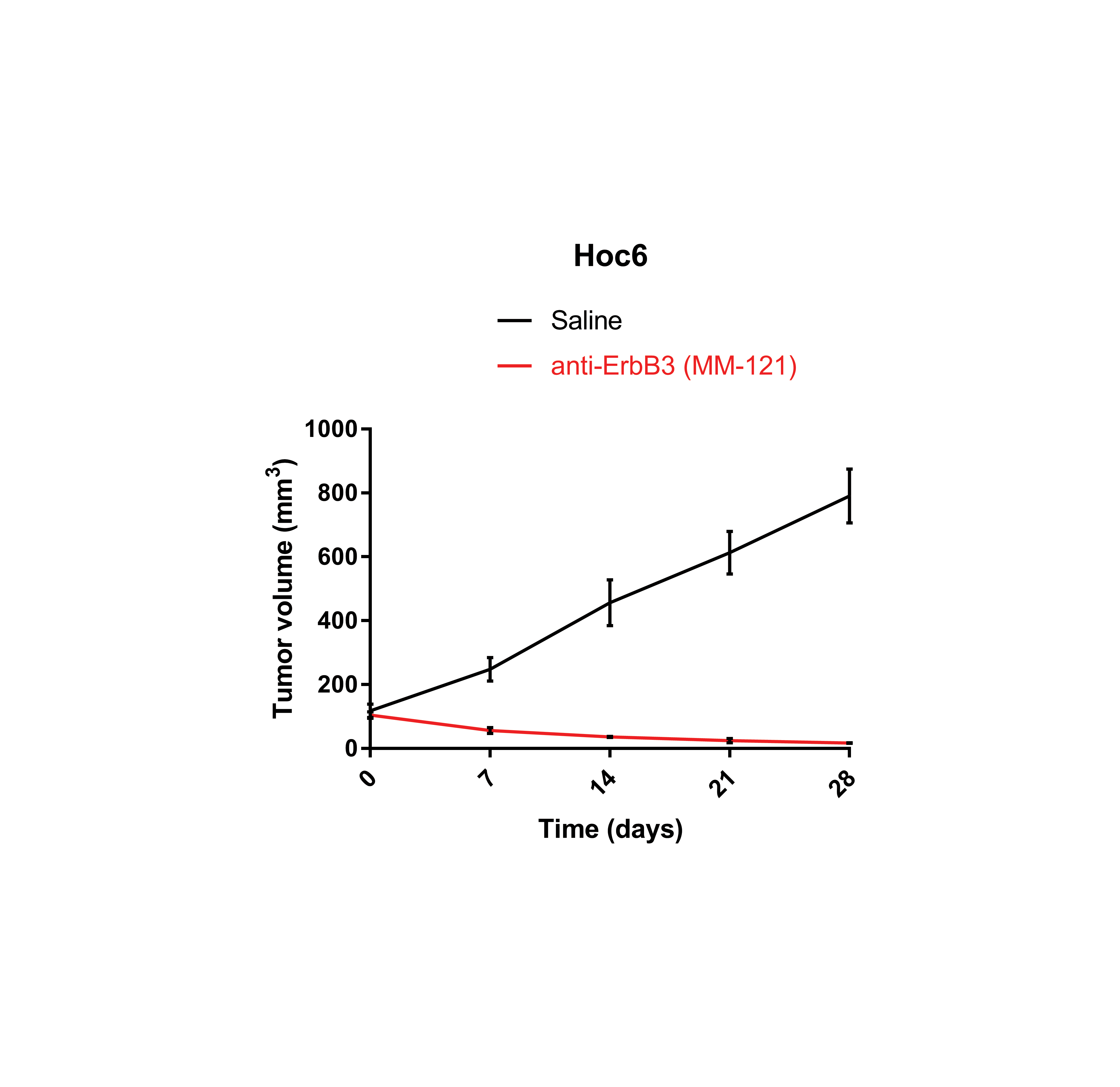

Supplement: Supplementary file 3 — Supplemental Figure 2 [file 41419_2017_29_MOESM3_ESM.png]

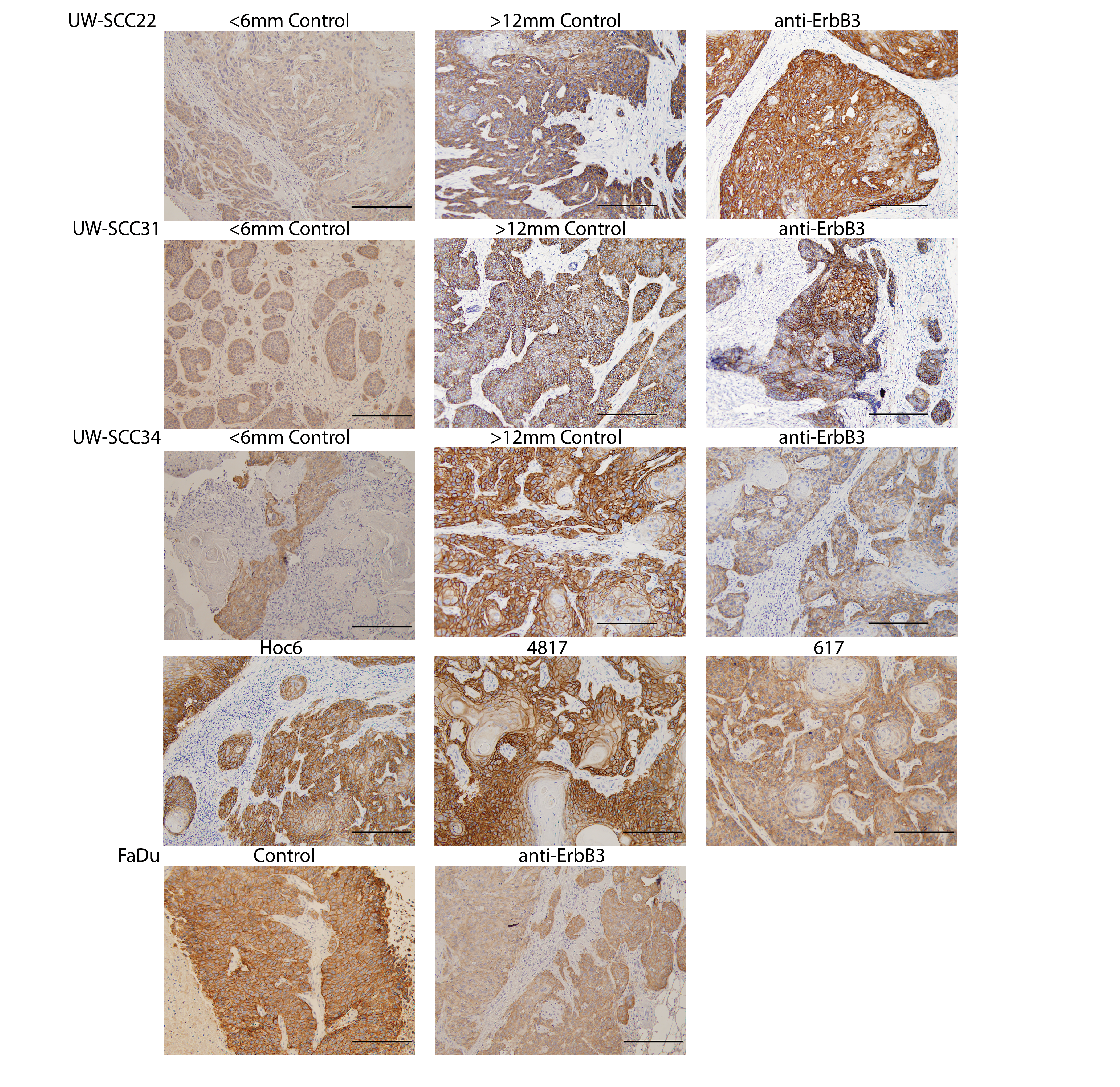

Supplement: Supplementary file 4 — Supplemental Figure 3 [file 41419_2017_29_MOESM4_ESM.png]

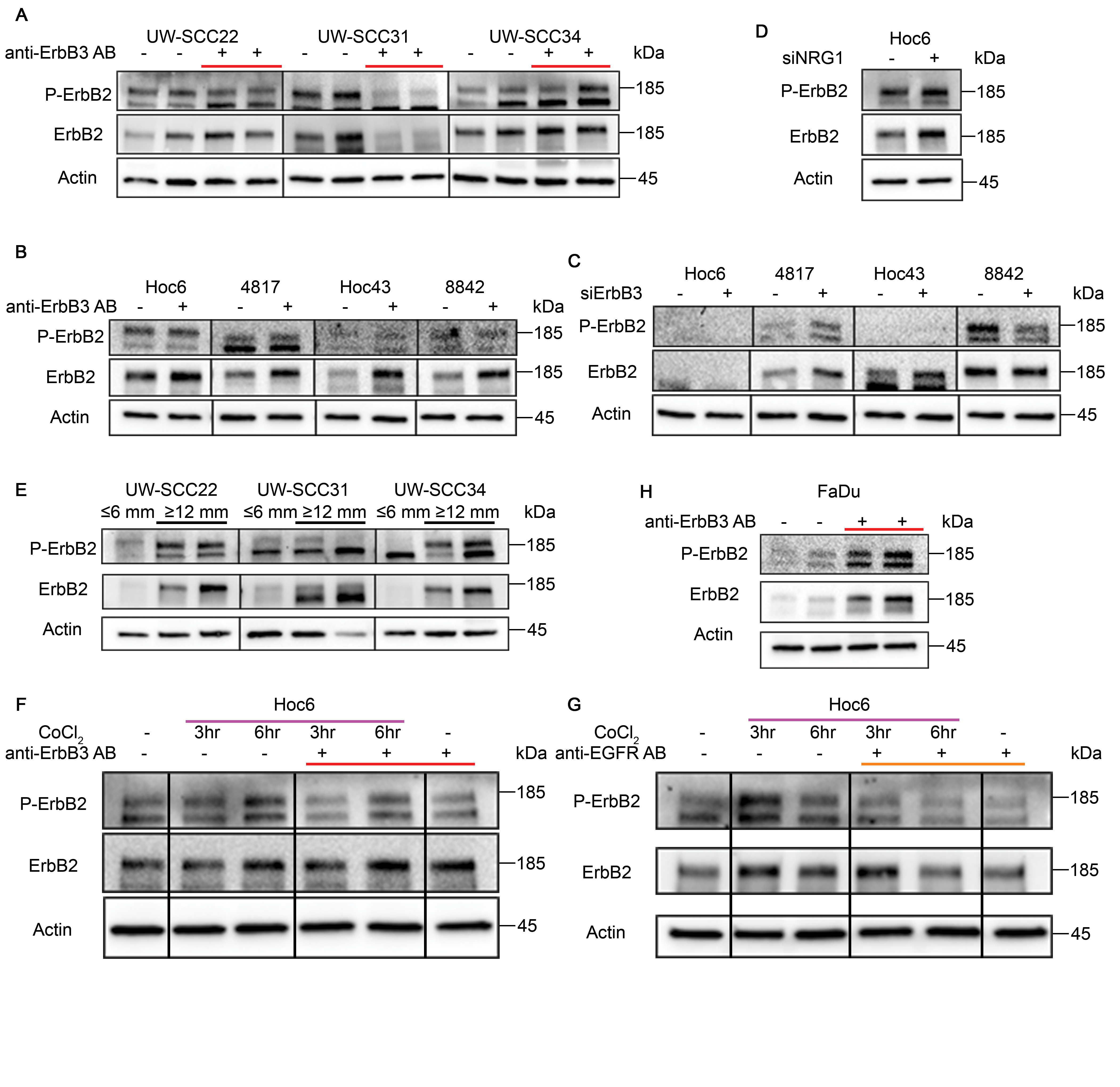

Supplement: Supplementary file 5 — Supplemental Figure 4 [file 41419_2017_29_MOESM5_ESM.png]

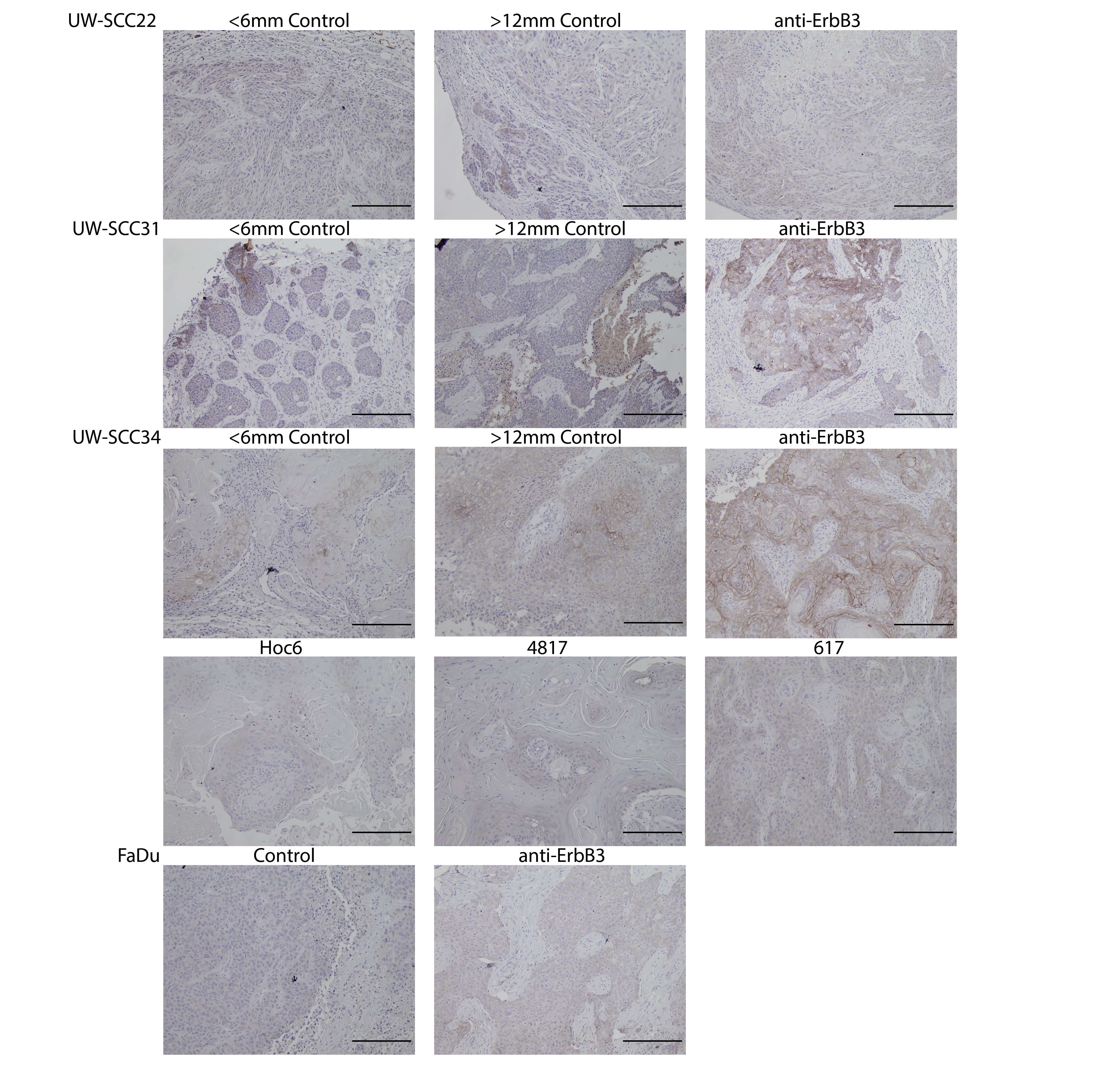

Supplement: Supplementary file 6 — Supplemental Figure 5 [file 41419_2017_29_MOESM6_ESM.png]

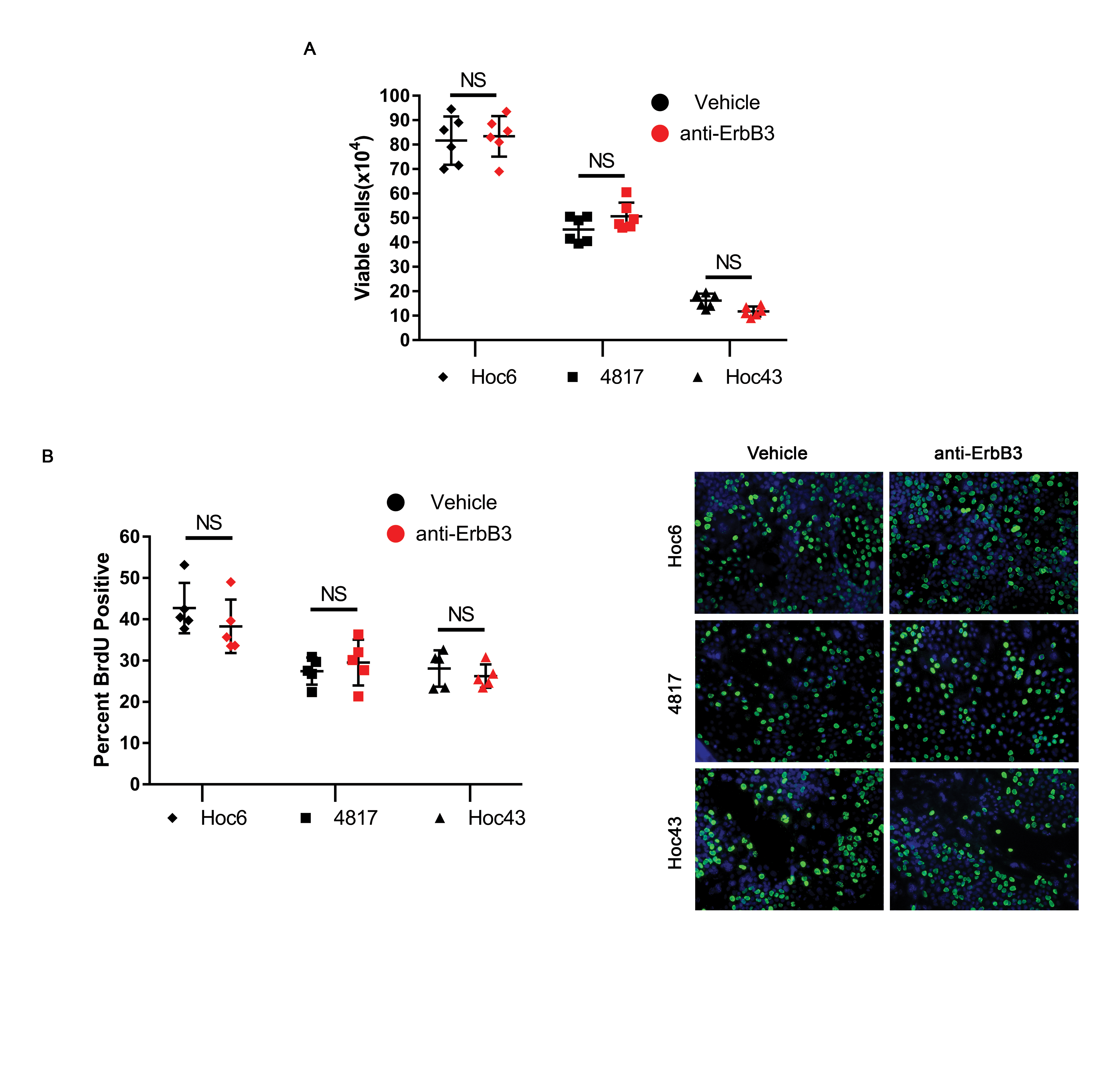

Supplement: Supplementary file 7 — Supplemental Figure 6 [file 41419_2017_29_MOESM7_ESM.png]

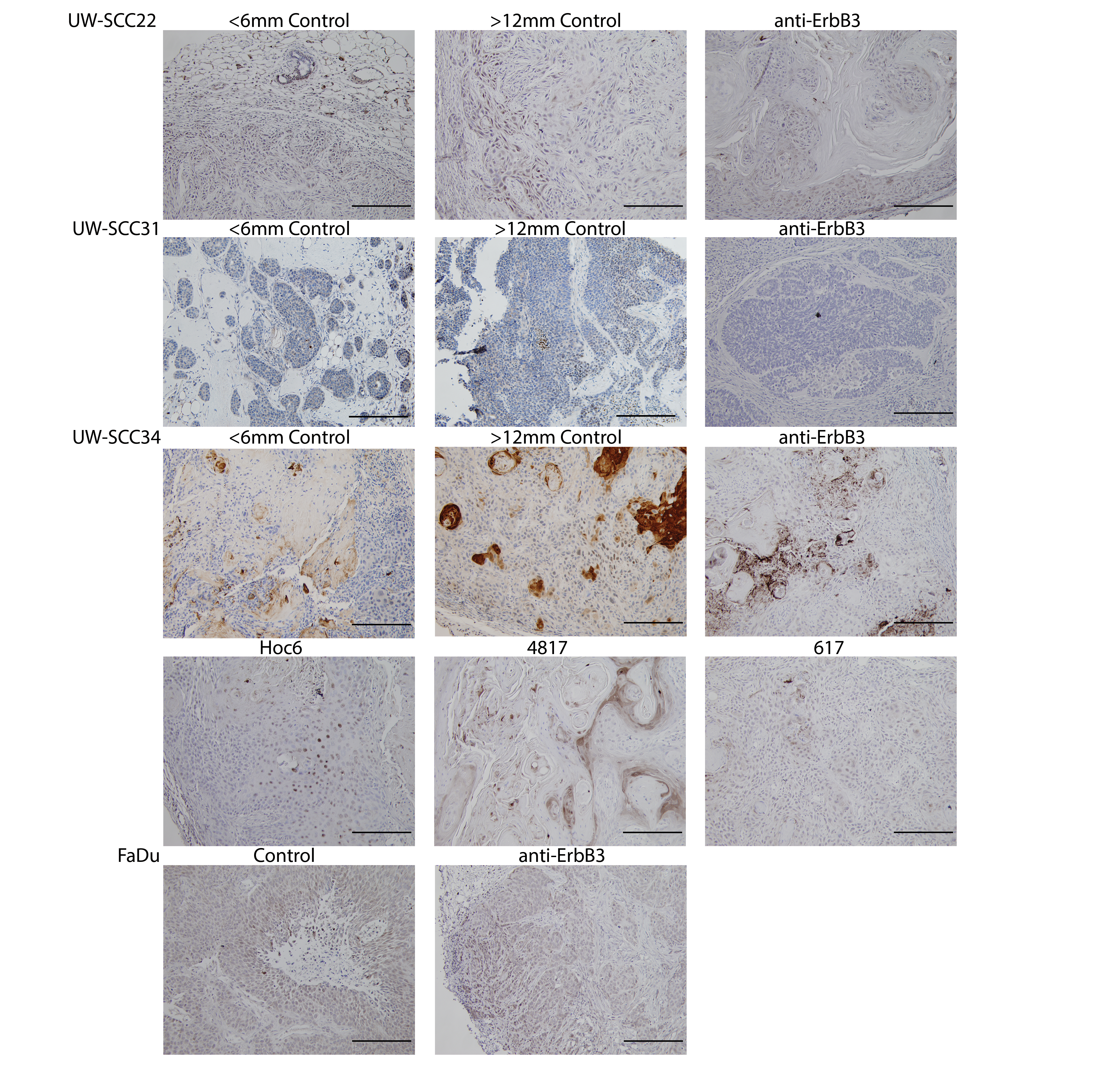

Supplement: Supplementary file 8 — Supplemental Figure 7 [file 41419_2017_29_MOESM8_ESM.png]

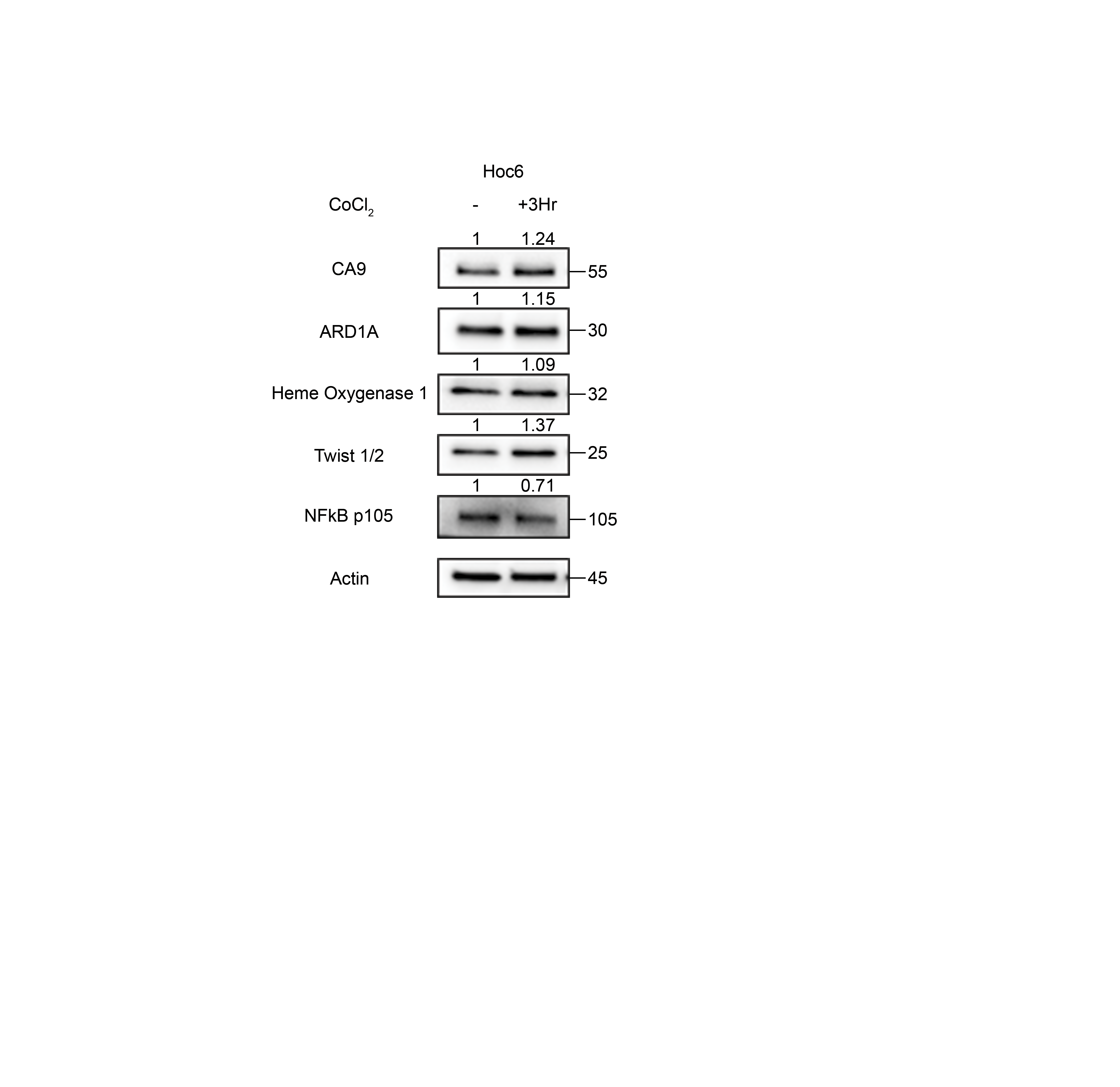

Supplement: Supplementary file 9 — Supplemental Figure 8 [file 41419_2017_29_MOESM9_ESM.png]

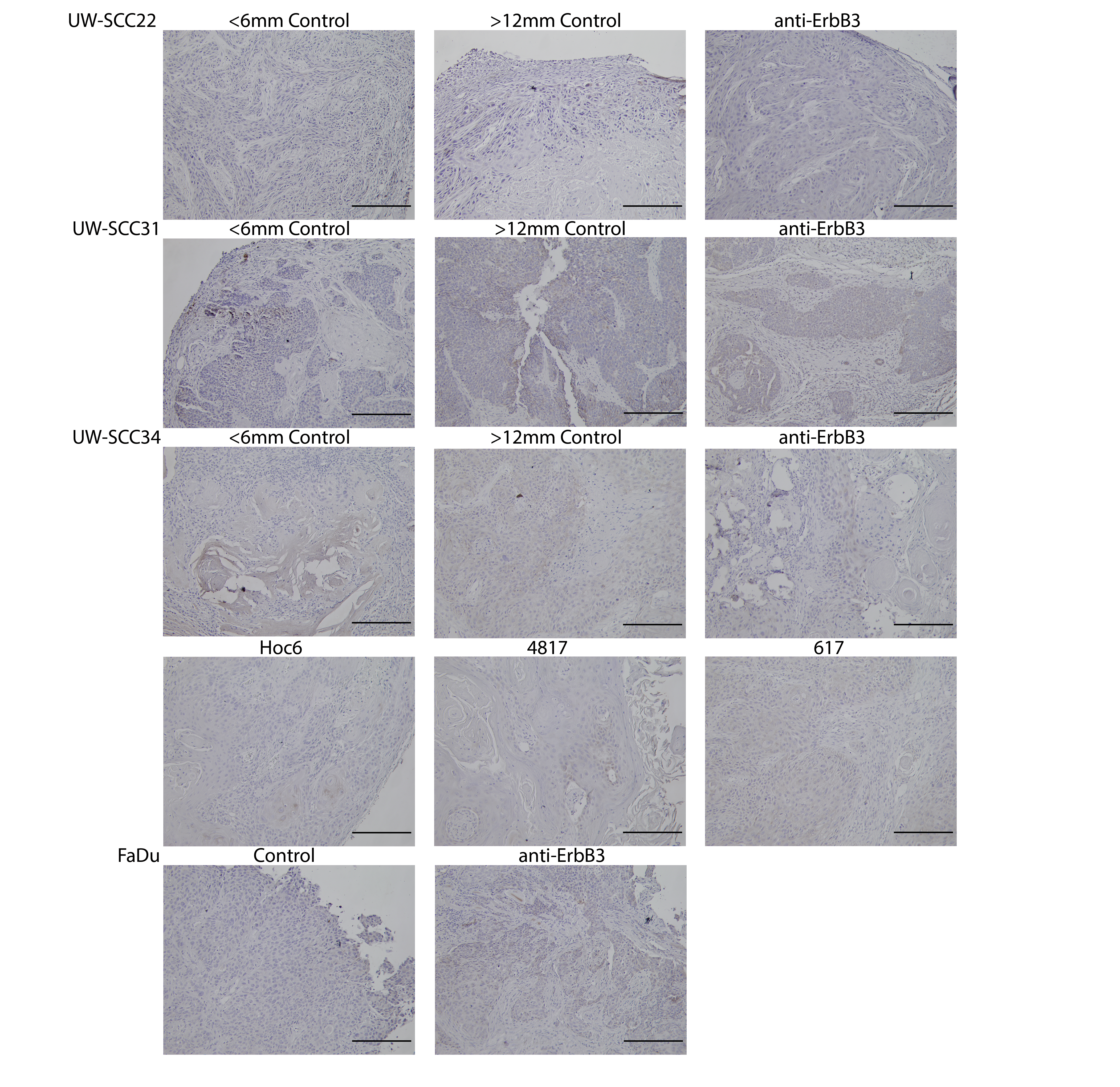

Supplement: Supplementary file 10 — Supplemental Figure 9 [file 41419_2017_29_MOESM10_ESM.png]
